# Age at type 2 diabetes onset, HOMA-derived indices and risks of diabetic retinopathy: a real-world cross-sectional study

**DOI:** 10.3389/fendo.2026.1772578

**Published:** 2026-05-11

**Authors:** Yewen Lin, Qiuju Zhou, Minghua Fan, Zihui Liu, Lisha Ni

**Affiliations:** Department of ophthalmology, The First Affiliated Hospital of Lishui University, Lishui People's Hospital, Lishui, Zhejiang, China

**Keywords:** diabetic retinopathy, HOMA-β, insulin resistance, type 2 diabetes mellitus, β-cell function

## Abstract

**Background:**

The roles of insulin resistance (IR) and β-cell dysfunction in diabetic retinopathy (DR) remain controversial, and whether age at type 2 diabetes mellitus (T2DM) onset modifies these associations is unclear. This study aimed to evaluate the associations of HOMA-IR and HOMA-β with DR risk, stratified by age at diagnosis.

**Methods:**

In this real-world cross-sectional study, we analyzed data from 6,996 patients with T2DM from Lishui People’s Hospital, China. Participants were categorized by age at onset (<65 vs. ≥65 years) and HOMA indices (HOMA-β <66 vs. ≥66; HOMA-IR <5 vs. ≥5). Multivariable logistic regression was used to assess adjusted odds ratios (aORs) for DR.

**Results:**

A total of 1,360 DR cases were identified in 6996 participants. Low HOMA-β was significantly associated with higher DR odds, especially in the younger-onset group (aOR = 2.97, 95% CI: 2.24–3.99). High HOMA-IR was associated with increased DR odds in younger-onset participants (aOR = 3.14, 95% CI: 2.31–4.33). Notably, among participants with high HOMA-β or low HOMA-IR, age at T2DM onset did not significantly modify the association with DR. Subgroup analyses showed strong associations in those with renal dysfunction or not using lipid-lowering agents.

**Conclusion:**

Both β-cell dysfunction and insulin resistance were independently associated with increased odds of diabetic retinopathy in younger-onset type 2 diabetes. Age at onset significantly modified these associations, supporting age-stratified screening and tailored management strategies.

## Introduction

Type 2 diabetes mellitus (T2DM) is characterized by two fundamental pathophysiological defects: insulin resistance and β-cell dysfunction (1, 2). While these metabolic abnormalities are well-established drivers of hyperglycemia, their contributions to the development of diabetic complications, particularly microvascular complications, remain incompletely understood (3, 4). The Homeostasis Model Assessment (HOMA) provides a valuable tool for quantifying these processes, with HOMA-IR measuring insulin resistance and HOMA-β estimating β-cell function (5, 6).

Among diabetes complications, diabetic retinopathy (DR) represents a particularly sight-threatening microvascular complication that remains a leading cause of blindness worldwide (7–9). Although chronic hyperglycemia has been considered as the primary risk factor for DR, emerging evidence suggested that the underlying metabolic defects of T2DM, insulin resistance and β-cell dysfunction, may independently contribute to its pathogenesis through various mechanisms, including inflammation, oxidative stress, and vascular endothelial dysfunction (10–13). Recent studies have emphasized that HOMA-derived indices serve as reliable surrogate measures for β-cell function and insulin resistance in both clinical and epidemiological settings. HOMA-β reflects residual pancreatic β-cell activity, while HOMA-IR quantifies the degree of insulin resistance, both of which are essential for understanding the heterogeneity of T2DM pathophysiology. This study aimed to elucidate the associations of HOMA-IR and HOMA-β with DR in a large cohort of T2DM patients, with specific focus on effect modification by age at diabetes onset.

## Methods

### Study design and population

**Figure 1:**
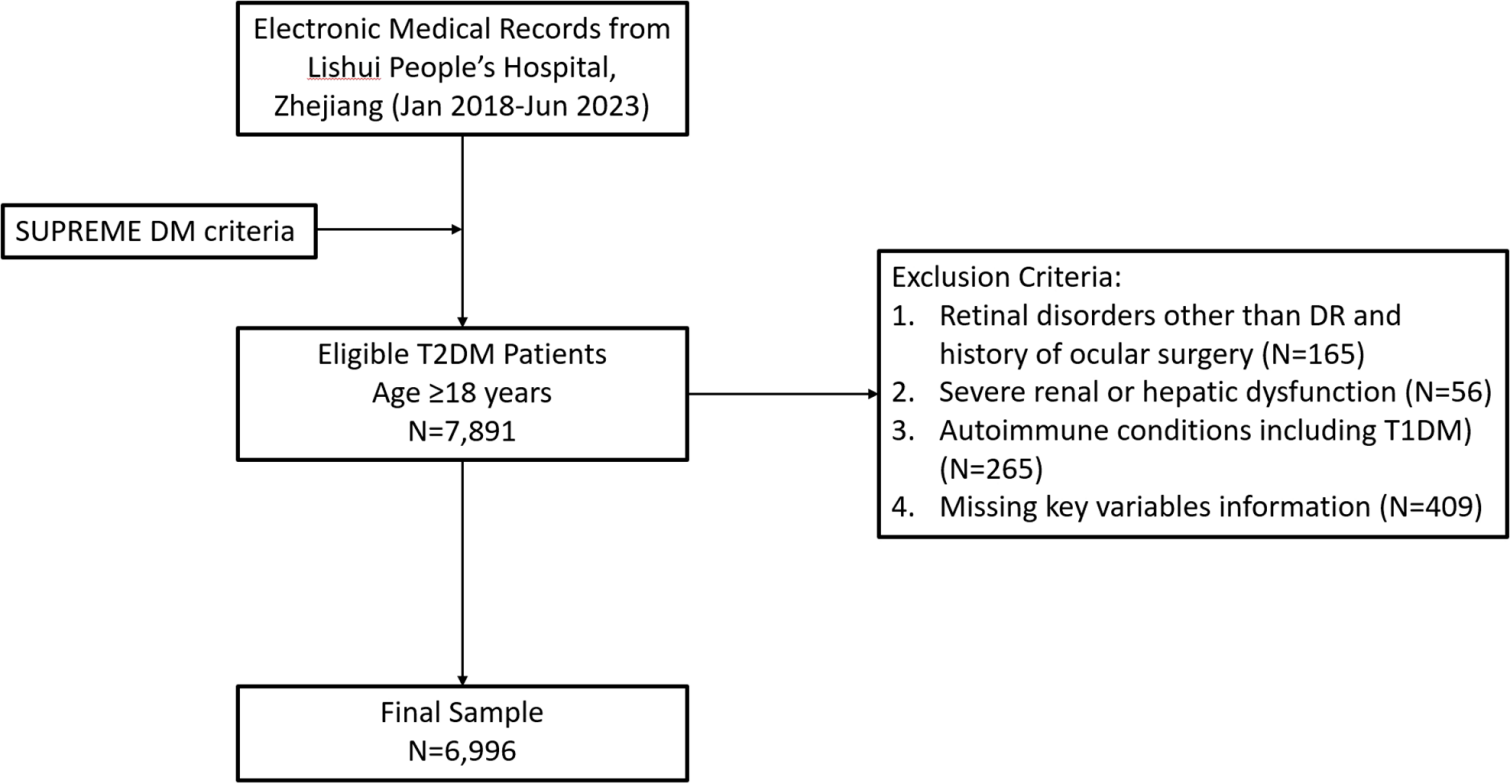
Flowchart.

This study constituted a real-world, cross-sectional analysis drawing upon electronic medical records from Lishui People’s Hospital, Zhejiang Province, China, covering the period from January 2018 to June 2023. The study population was composed of adult patients with a confirmed diagnosis of T2DM. Exclusion criteria encompassed: pre-existing non-diabetic retinal pathology, prior ocular surgical procedures, severe renal insufficiency (eGFR <30 mL/min/1.73 m²) or documented hepatic impairment, autoimmune disorders (including type 1 diabetes), current use of insulin or insulin secretagogues (because exogenous insulin and secretagogue therapy materially alter fasting insulin concentrations, thereby reducing the interpretability of HOMA-derived indices), and instances of critical missing data (specifically, fasting glucose, fasting insulin, or retinopathy assessment outcomes). From an initial pool of 7,891 screened patients, 6,996 patients met all eligibility requirements and were included in the final analysis (**Figure 1**). The study received ethical approval from the Institutional Review Board of Lishui People’s Hospital (2024-025-01), and the need for individual informed consent was waived due to the retrospective and anonymized nature of the data analysis.

### Clinical and laboratory assessments

All data was procured during standard clinical care encounters. Following an overnight fast of at least 8 hours, antecubital venous blood samples were collected. Standardized instruments were employed to measure height and weight, with values documented to the nearest 0.1 cm and 0.1 kg, respectively; body mass index (BMI) was subsequently derived. Resting brachial blood pressure was recorded in duplicate using an automated sphygmomanometer after the participant had been seated for five minutes, and the mean of these two readings was utilized.

A thorough review of patient records furnished data on comorbid conditions such as hypertension and dyslipidemia, and detailed medication use. The laboratory evaluation comprised fasting plasma glucose (FPG), glycated hemoglobin (HbA1c), a comprehensive lipid panel (total cholesterol, LDL-C, HDL-C, triglycerides), serum creatinine, and fasting serum insulin levels. FPG and lipid parameters were determined via enzymatic assays, HbA1c was quantified using high-performance liquid chromatography (HPLC), and serum insulin was measured via an electrochemiluminescence immunoassay (ECLIA).

### Calculation of HOMA indices and group stratification

β-cell function and insulin resistance were estimated using the Homeostasis Model Assessment, calculated as follows: HOMA-IR = (Fasting Insulin (μU/mL) × Fasting Glucose (mmol/L))/22.5 and HOMA-β = (20 × Fasting Insulin (μU/mL))/(Fasting Glucose (mmol/L) - 3.5) (14). HOMA-β was dichotomized using a threshold of 66, while HOMA-IR utilized a cutoff of 5.0, thereby defining subgroups with relatively preserved versus impaired β-cell function and lower versus higher insulin resistance, respectively. These were pragmatic thresholds for categorical presentation rather than universal biological cutoffs. Age at diabetes onset was dichotomized as <65 years (younger-onset) or ≥65 years (older-onset), a stratification chosen to reflect potentially distinct pathophysiological phenotypes.

The diagnosis of T2DM was established in accordance with the SUPREME-DM criteria, which synthesizes diagnostic codes (ICD-10-CM), laboratory results, and pharmacotherapy data. Confirmation required: (a) at least one inpatient ICD-10 code for T2DM; (b) two or more outpatient ICD codes occurring on distinct dates within a 24-month window; or (c) a combination of outpatient codes alongside corroborative laboratory evidence (FPG ≥7.0 mmol/L, random glucose ≥11.1 mmol/L, or HbA1c ≥6.5%) or a documented prescription for antihyperglycemic agents.

### Assessment of diabetic retinopathy

Retinal status was evaluated from digital fundus photographs obtained with a Canon CR-2 AF retinal camera. The grading of DR severity adhered to the classification guidelines set forth by the Early Treatment Diabetic Retinopathy Study (ETDRS). The prespecified primary outcome was prevalent diabetic retinopathy of any severity. Although ETDRS grading data were available, the total number of moderate-to-severe nonproliferative and proliferative DR cases was small (N = 64), which limited statistical power for severity-specific analyses. To ensure accuracy and minimize bias, all fundus images were assessed independently by two ophthalmologists who were blinded to the clinical and biochemical data of the participants. Any discordance in grading was resolved through review and consensus with a third senior ophthalmologist.

### Statistical analyses

Continuous variables are presented as mean ± standard deviation and categorical variables as counts and percentages. Group comparisons for baseline characteristics used the Student t test or analysis of variance for continuous variables and the chi-square test for categorical variables. Because this was an observational study and the exposure-defined groups were not expected to be balanced at baseline, **Table 1** is descriptive and confounding was addressed in multivariable models rather than by design. Associations between HOMA indices and prevalent DR were evaluated using multivariable logistic regression and are reported as odds ratios (ORs) with 95% confidence intervals (CIs). We prespecified Model A without HbA1c to estimate the overall association and Model B with HbA1c to estimate the association not explained by contemporaneous glycemia. Both models adjusted for sex, BMI, systolic blood pressure, LDL-cholesterol, triglycerides, eGFR, diabetes duration, and use of antihypertensive, lipid-lowering, and glucose-lowering medications. For joint analyses of HOMA-β and HOMA-IR, the reference category was high HOMA-β and low HOMA-IR. We assessed collinearity using variance inflation factors, tested multiplicative interactions between HOMA-β and HOMA-IR and between age at onset and each HOMA index, and performed sensitivity analyses using quartiles, restricted cubic splines, duration strata (<5 years, 5–10 years, >10 years), and alternative age-at-onset definitions. Subgroup analyses are reported with subgroup sample sizes and interaction p values and are interpreted cautiously when estimates are imprecise. Statistical analyses were performed using R version 4.0.3, and a two-sided *P* value <0.05 was considered statistically significant.

**Table 1:** Baseline characteristics based on different ages at DM onset and HOMA-beta.

	Overall	Age at DM onset < 65	Age at DM onset ≥ 65	Age at DM onset < 65	Age at DM onset ≥ 65
		HOMA-beta < 66	HOMA-beta < 66	HOMA-beta ≥ 66	HOMA-beta ≥ 66
No. of participants	6996	3042	433	2964	557
Age (years)	58.98 (13.21)	55.37 (11.95)	76.31 (5.38)^*^	57.00 (11.89)	75.75 (5.53)^#^
BMI (kg/m^2^)	23.85 (2.99)	23.77 (2.91)	23.50 (2.59)	23.98 (3.14)	23.79 (2.80)
Duration of DM	8.01 (6.33)	8.03 (6.24)	5.47 (4.74) ^*^	8.76 (6.71)	5.92 (4.72) ^#^
Systolic blood pressure (mmHg)	123.32 (20.62)	122.78 (20.13)	123.92 (21.78)	123.12 (20.48)	126.80 (22.73)^#^
Diastolic blood pressure (mmHg)	77.25 (9.30)	77.79 (9.61)	75.33 (8.22)^*^	77.26 (9.23)	75.76 (8.45)
Fasting plasma glucose (mmol/L)	8.40 (2.95)	9.80 (3.08)	9.58 (3.47)^*^	7.07 (1.98)	6.97 (1.93)
FINS (IU/L)	24.15 (55.61)	9.84 (4.39)	10.3 (4.86)	37.6 (72.3)	43.4 (94.7)^#^
HOMA-IR	8.13 (12.0)	3.11 (1.13)	3.08 (1.15)	13.1 (15.3)	13.7 (15.7)
HOMA-beta	122.5 (175)	81.9 (107)	95.2 (131)^*^	158 (212)	179 (230)^#^
HbA1c (%)	9.05 (2.26)	9.39 (2.17)	9.53 (2.51)	8.66 (2.19)	8.83 (2.43)
eGFR (mL/min/1.73 m²)	95.33 (20.64)	100.59 (18.04)	79.22 (17.76)^*^	95.85 (20.58)	76.35 (19.03)^#^
LDL-cholesterol (mmol/L)	3.09 (0.94)	3.17 (0.96)	3.13 (0.95)	3.03 (0.93)	2.94 (0.85)
Triglycerides (mmol/L)	1.85 (1.71)	2.01 (2.04)	1.52 (0.82)^*^	1.77 (1.49)	1.61 (1.13)
HDL-cholesterol (mmol/L)	1.14 (0.30)	1.14 (0.29)	1.17 (0.33)	1.14 (0.30)	1.13 (0.30)
Total cholesterol (mmol/L)	4.72 (1.08)	4.83 (1.12)	4.73 (1.03)	4.65 (1.05)	4.55 (0.97)
Sex					
Male	3927 (56.1)	1821 (59.9)	221 (51.0)^*^	1610 (54.3)	275 (49.4)^#^
Female	3069 (43.9)	1221 (40.1)	212 (49.0)^*^	1354 (45.7)	282 (50.6)^#^
Anti-diabetes medications (%)	1291 (18.5)	501 (16.5)	71 (16.4)	610 (20.6)	109 (19.6)
Metformin	803 (11.5)	291 (9.6)	28 (6.5)	408 (13.8)	76 (13.6)
DPP4i	201 (2.9)	84 (2.8)	20 (4.6)	88 (3.0)	9 (1.6)
SGLT2i	36 (0.5)	14 (0.5)	6 (1.4)	15 (0.5)	1 (0.2)
TZD	63 (0.9)	25 (0.8)	6 (1.4)	31 (1.0)	1 (0.2)
GLP-1RA	396 (5.7)	168 (5.5)	33 (7.6)	165 (5.6)	30 (5.4)
Lipid-lowering medications (%)	560 (8.0)	244 (8.0)	47 (10.9)	217 (7.3)	52 (9.3)
Anti-hypertension medications (%)	661 (9.4)	276 (9.1)	40 (9.2)	293 (9.9)	52 (9.3)

Data were mean (SD) or number (%).

^*^ *P* < 0.05, Age at DM onset < 65 & HOMA- beta < 66 vs. Age at DM onset ≥ 65 & HOMA-beta < 66.

^#^ *P* < 0.05, Age at DM onset < 65 & HOMA- beta ≥ 66 vs. Age at DM onset >=65 & HOMA-beta ≥ 66.

## Results

### Participant selection and baseline characteristics

A total of 6,996 participants with type 2 diabetes were included in the final analysis. The baseline characteristics, stratified by age at diabetes onset and HOMA indices, are presented in **Table 1**. The older-onset group was smaller than the younger-onset group because, in this hospital-based cohort, most patients had been diagnosed before age 65. As expected in an observational study stratified by age at onset and metabolic phenotype, sex, BMI, fasting plasma glucose, HbA1c, renal function, and medication use differed across groups (**Table 1**). These imbalances motivated multivariable adjustment and sensitivity analyses rather than simple interpretation of crude group comparisons. The overall mean HOMA-IR and HOMA-β were 8.13 and 122.5, respectively.

Participants were categorized into four groups: younger-onset diabetes (<65 years) with low HOMA-β (<66, n=3,042), older-onset diabetes (≥65 years) with low HOMA-β (n=433), younger-onset diabetes with high HOMA-β (≥66, n=2,964), and older-onset diabetes with high HOMA-β (n=557).

Significant differences were observed across all metabolic parameters. Participants with low HOMA-β exhibited markedly higher fasting glucose (9.80 vs 7.07 mmol/L in younger-onset; 9.58 vs 6.97 mmol/L in older-onset) and HbA1c levels (9.39% vs 8.66% in younger-onset; 9.53% vs 8.83% in older-onset) compared to those with high HOMA-β, regardless of age group. Renal function, as measured by eGFR, was significantly lower in older-onset groups and further impaired in those with low HOMA-β. Lipid profiles and blood pressure measures also varied significantly across groups, with consistently less favorable parameters observed in the low HOMA-β groups.

### Association between HOMA indices and Diabetic Retinopathy in different ages at T2DM onset

A total of 1,360 prevalent DR cases were identified among 6,996 participants. The associations between HOMA indices, age at onset, and diabetic retinopathy are summarized in **Table 2**. After multivariable adjustment for sex, BMI, systolic blood pressure, LDL-cholesterol, triglyceride levels, HbA1c, eGFR, and the use of antihypertensive, lipid-lowering, and glucose-lowering medications, low HOMA-β (<66) was strongly associated with increased odds of DR, particularly in the younger-onset group (aOR = 2.97, 95% CI: 2.24-3.99) compared to the reference group (older-onset, high HOMA-β). A similar but attenuated association was observed in the older-onset, low HOMA-β group (aOR = 2.75, 95% CI: 2.06-3.71). Notably, among participants with preserved β-cell function (HOMA-β ≥66), age at T2DM onset did not significantly modify the risk of diabetic retinopathy. The comparison between younger and older-onset groups within this high HOMA-β stratum showed no statistically significant difference in DR (aOR = 0.94, 95% CI: 0.63-1.39).

**Table 2:** Odds ratios of diabetic retinopathy based on participants with different ages at DM onset, different levels of HOMA-beta and HOMA-IR.

	No. of participants	No. of DR case	Crude model	Model 1	Model 2
Age at DM onset ≥ 65 and HOMA-beta ≥ 66	557	67	1	1	1
Age at DM onset ≥ 65 and HOMA-beta < 66	433	49	0.93 (0.63, 1.38)	0.99 (0.66, 1.46)	0.95 (0.64, 1.41)
Age at DM onset < 65 and HOMA-beta < 66	3042	604	1.81 (1.39, 2.39)	1.70 (1.28, 2.28)	1.67 (1.26, 2.25)
Age at DM onset < 65 and HOMA-beta ≥ 66	2964	640	2.01 (1.55, 2.66)	1.74 (1.32, 2.33)	1.79 (1.35, 2.40)
Age at DM onset ≥ 65 and HOMA-IR < 5	483	55	1	1	1
Age at DM onset < 65 and HOMA-IR < 5	3021	585	1.87 (1.40, 2.53)	1.71 (1.26, 2.34)	1.72 (1.27, 2.36)
Age at DM onset < 65 and HOMA-IR ≥ 5	2985	659	2.20 (1.66, 2.99)	1.92 (1.42, 2.63)	1.84 (1.36, 2.53)
Age at DM onset ≥ 65 and HOMA-IR ≥ 5	507	61	1.06 (0.72, 1.57)	1.08 (0.73, 1.61)	1.00 (0.68, 1.49)
HOMA-beta ≥ 66 and HOMA-IR < 5	1326	244	1	1	1
HOMA-beta ≥ 66 and HOMA-IR ≥ 5	2195	463	1.19 (1.00, 1.41)	1.12 (0.94, 1.35)	1.06 (0.88, 1.27)
HOMA-beta < 66 and HOMA-IR < 5	2178	396	0.99 (0.83, 1.18)	1.01 (0.84, 1.22)	0.96 (0.80, 1.16)
HOMA-beta < 66 and HOMA-IR ≥ 5	1297	257	1.10 (0.90, 1.33)	1.12 (0.91, 1.37)	1.00 (0.81, 1.23)

Model 1 was adjusted for sex, BMI, blood pressure, LDL-cholesterol, triglyceride levels, eGFR, diabetes duration, and the use of antihypertensive, lipid-lowering, glucose-lowering medications and duration of DM.

Model 2 was adjusted for all variables in Model 1 plus HbA1c.

Similarly, high HOMA-IR (≥ 5) was associated with significantly increased DR odds in younger-onset diabetes (aOR = 3.14, 95% CI: 2.31-4.33) compared to the reference group (older-onset, low HOMA-IR). However, among participants with low insulin resistance (HOMA-IR < 5), the effect of age on DR risk was substantially attenuated and not statistically significant (aOR = 1.00, 95% CI: 0.68-1.49 for older-onset vs. younger-onset with low HOMA-IR). When both HOMA indices were considered simultaneously, the combination of low HOMA-β and high HOMA-IR yielded a point estimate modestly above unity (aOR = 1.08, 95% CI: 0.88–1.33) relative to the reference category of high HOMA-β and low HOMA-IR, but the confidence interval included the null. This absence of a clear synergistic effect should be interpreted in the context of moderate collinearity between HOMA-β and HOMA-IR and of possible attenuation from adjusting for HbA1c, which may lie on the causal pathway.

### Subgroup and interaction analyses

Stratified analyses revealed significant effect modification by several baseline characteristics (**Table 3**, **Supplementary Table 1**). The association between low HOMA-β and DR was particularly pronounced in participants with impaired renal function (eGFR <60 mL/min/1.73 m²: aOR = 3.36, 95% CI: 1.74-6.79) and those not using lipid-lowering medications (aOR = 1.79, 95% CI: 1.36-2.40). For HOMA-IR, the strongest associations with DR were observed in younger-onset participants with preserved renal function (eGFR ≥90 mL/min/1.73 m²: aOR = 1.99, 95% CI: 1.24–3.40) and those using antihypertensive medications (aOR = 8.14, 95% CI: 2.43–50.7). The very wide confidence interval in the antihypertensive subgroup reflects sparse data and should be interpreted with caution.

**Table 3:** Subgroup analysis on interactions of odds of diabetic retinopathy among participants with different baseline characteristics of ages at DM onset and HOMA-beta.

	Age at DM onset ≥ 65 and HOMA-beta ≥ 66	Age at DM onset < 65 and HOMA-beta < 66	Age at DM onset ≥ 65 and HOMA-beta < 66	Age at DM onset < 65 and HOMA-beta ≥ 66	HOMA-beta as a continuous variable Log_10_
Sex					
Male	1	2.51 (1.63, 4.03)	1.71 (0.96, 3.06)	2.49 (1.62, 3.98)	1.00 (1.00, 1.00)*
Female	1	1.23 (0.85, 1.82)	0.62 (0.34, 1.08)	1.32 (0.91, 1.93)	1.00 (1.00, 1.00)*
Estimated GFR, mL/min/1.73 m^2^					
≥ 90	1	1.44 (0.91, 2.41)	1.11 (0.56, 2.20)	1.49 (0.94, 2.50)	1.00 (1.00, 1.00)*
60-89	1	1.76 (1.14, 2.78)	0.76 (0.41, 1.40)	1.68 (1.10, 2.62)	1.00 (1.00, 1.00)
< 60	1	3.03 (1.51, 6.33)	1.35 (0.57, 3.15)	2.80 (1.50, 5.50)	1.00 (1.00, 1.00)
Lipid-lowering medications					
No use	1	1.70 (1.27, 2.32)	0.98 0.64, 1.49)	1.74 (1.30, 2.37)	1.00 (1.00, 1.00)*
Use	1	1.65 (0.64, 4.87)	0.99 (0.28, 3.49)	1.77 (0.70, 5.12)	1.00 (1.00, 1.00)
Antihypertensive medications					
No use	1	1.71 (1.27, 2.34)	1.07 (0.70, 1.61)	1.78 (1.33, 2.43)	1.00 (1.00, 1.00)*
Use	1	1.69 (0.72, 4.50)	0.48 (0.10, 1.88)	1.56 (0.68, 4.09)	1.00 (1.00, 1.00)
Glucose-lowering medications	1	3.22 (1.48, 8.07)	1.82 (0.62, 5.49)	3.04 (1.42, 7.55)	1.00 (1.00, 1.00)
GLP-1RA	1	2.88 (1.21, 8.51)	1.09 (0.15, 5.42)	3.41 (1.46, 9.96)	1.00 (1.00, 1.00)*
DPP4i	1	2.67 (0.45, 51.0)	0.42 (0.01, 11.5)	2.35 (0.40, 45.0)	1.00 (1.00, 1.00)
SGLT2i	–	1	0.27 (0.01, 2.28)	1.17 (0.27, 5.19)	1.00 (1.00, 1.00)
TZD	–	1	0.63 (0.03, 0.75)	1.10 (0.33, 3.87)	1.00 (1.00, 1.00)
Metformin	1	10.6 (2.17, 191)	2.90 (0.35, 60.5)	8.98 (1.82, 162)	1.00 (1.00, 1.00)

Model was adjusted for sex, BMI, blood pressure, LDL-cholesterol, triglyceride levels, eGFR, and the use of antihypertensive, lipid-lowering, glucose-lowering medications, and duration of DM other than the variables for stratification. All *P* for interaction > 0.05. * indicates P < 0.05.

## Discussion

This large cross-sectional study demonstrated that both low HOMA-β and high HOMA-IR were independently associated with increased odds of diabetic retinopathy, with particularly strong associations observed in younger-onset type 2 diabetes.

Age significantly influences the presentation of the two core pathophysiological defects in T2DM including insulin resistance and β-cell dysfunction (15, 16). Younger-onset diabetes is often characterized by a more pronounced loss of β-cell function, while older-onset diabetes may be predominantly influenced by IR (17, 18). HOMA provides a practical method to estimate these traits, with HOMA-IR quantifying insulin resistance and HOMA-β reflecting β-cell function (14, 19), offering crucial insights into the heterogeneous metabolic profiles across different age groups in T2DM.

We found the robust association between low HOMA-β and DR risk, especially in younger-onset diabetes, underscores the critical role of β-cell function in the pathogenesis of diabetic microvascular complications. Younger individuals with diabetes typically experience more rapid β-cell decline and consequently more aggressive disease progression (20, 21). These cross-sectional associations are consistent with the hypothesis that poor β-cell function contributes to retinopathy through sustained hyperglycemia and possibly through direct effects on retinal vascular integrity. The significantly higher glucose and HbA1c levels in the low HOMA-β groups are compatible with this interpretation. Notably, the association between low HOMA-β and DR persisted after adjustment for HbA1c (Model B), suggesting that part of the association may operate through pathways other than contemporaneous glycemic control alone.

The differential impact of HOMA-IR based on age at onset is particularly noteworthy. While high HOMA-IR significantly increased DR risk in younger-onset diabetes, no association was observed in older-onset diabetes. This divergence may reflect distinct pathophysiological phenotypes: younger participants may harbor more pronounced lipotoxicity and inflammatory signaling associated with insulin resistance, whereas older participants may have developed compensatory mechanisms or present with different patterns of metabolic dysfunction (12, 22, 23). The stronger association observed in those with preserved renal function is consistent with the possibility that the relationship between insulin resistance and retinopathy is most readily detected before the onset of significant nephropathy.

The effect modification by renal function deserves special attention. The stronger association between low HOMA-β and DR in participants with impaired kidney function may reflect shared pathways of microvascular injury or the accumulation of toxic metabolites that exacerbate both renal and retinal damage (24–26). This finding highlighted the interconnected nature of diabetic complications and suggested that patients with concurrent renal impairment and β-cell dysfunction represent a particularly high-risk population requiring intensified monitoring and intervention.

Several limitations should be acknowledged. First, the cross-sectional design precludes causal inference; all reported associations should be understood as reflecting coexistence rather than temporal sequence. Second, residual confounding by incompletely measured factors, including lifestyle behaviors and genetic predisposition, cannot be excluded, although we adjusted for diabetes duration and other major covariates. Third, the use of HOMA indices rather than gold-standard measures such as the hyperinsulinemic-euglycemic clamp or intravenous glucose tolerance test may introduce measurement error. Fourth, the exclusion of patients receiving insulin or insulin secretagogues was necessary to preserve the validity of HOMA-derived indices, but it likely biases the sample toward patients with less advanced disease and may limit generalizability to the broader T2DM population. Fifth, the single-center setting may restrict external validity, though the large sample size enhances the statistical robustness of our findings. Future prospective studies should be conducted to determine whether these associations are causal and to validate the observed age-stratified patterns.

## Conclusion

Our study demonstrated that both β-cell dysfunction and insulin resistance were significantly associated with diabetic retinopathy, with particularly strong associations in younger-onset type 2 diabetes. These findings support age-stratified approaches to DR screening and suggest that preservation of β-cell function and improvement of insulin sensitivity warrant investigation as therapeutic targets beyond glycemic control alone, particularly in younger-onset type 2 diabetes.

## Data Availability

The original contributions presented in the study are included in the article/**Supplementary Material**. Further inquiries can be directed to the corresponding author.

## Supplementary material

Supplementary Table 1 Subgroup analysis on interactions of odds of diabetic retinopathy among participants with different baseline characteristics of ages at DM onset and HOMA-IR.

Supplementary Table 2 Multiplicative interaction between HOMA-IR and HOMA-β in relation to diabetic retinopathy.
