# Supplementary material for: Age at type 2 diabetes onset, HOMA-derived indices and risks of diabetic retinopathy: a real-world cross-sectional study
Source: Front Endocrinol (Lausanne). 2026 May 11;17:1772578. doi: 10.3389/fendo.2026.1772578 (PMC13199103; doi:10.3389/fendo.2026.1772578)

Supplementary Table 1. Subgroup analysis on interactions of odds of diabetic retinopathy among participants with different baseline characteristics of ages at DM onset and HOMA-IR

|  | Age at DM onset ≥ 65 and HOMA-IR < 5 | Age at DM onset < 65 and HOMA-IR < 5 | Age at DM onset < 65 and HOMA-IR ≥ 5 | Age at DM onset ≥ 65 and HOMA-IR ≥ 5 | HOMA-IR as a continuous variable |
| --- | --- | --- | --- | --- | --- |
| Sex |  |  |  |  |  |
| Male | 1 | 2.04 (1.35, 3.18) | 2.16 (1.43, 3.38) | 1.19 (0.67, 2.11) | 1.01 (1.00, 1.01) |
| Female | 1 | 1.36 (0.88, 2.17) | 1.64 (1.07, 2.61) | 0.96 (0.56, 1.66) | 1.01 (1.00, 1.02) |
| Estimated GFR, mL/min/1.73 m^2^ |  |  |  |  |  |
| ≥90 | 1 | 1.40 (0.87, 2.41) | 1.63 (1.00, 2.81) | 1.17 (0.59, 2.32) | 1.01 (1.01, 1.02) |
| 60-89 | 1 | 1.96 (1.23, 3.25) | 1.99 (1.24, 3.30) | 1.07 (0.59, 1.94) | 1.01 (1.00, 1.01) |
| <60 | 1 | 2.35 (1.16, 4.98) | 2.56 (1.27, 5.41) | 0.93 (0.40, 2.16) | 1.00 (0.99, 1.01) |
| Lipid-lowering medications |  |  |  |  |  |
| No use | 1 | 1.63 (1.20, 2.27) | 1.85 (1.35, 2.57) | 1.00 (0.66, 1.51) | 1.01 (1.00, 1.01) |
| Use | 1 | 2.78 (0.99, 9.97) | 2.95 (1.06, 10.6) | 2.48 (0.70, 10.2) | 1.02 (1.00, 1.04) |
| Antihypertensive medications |  |  |  |  |  |
| No use | 1 | 1.55 (1.14, 2.15) | 1.73 (1.27, 2.40) | 0.93 (0.61, 1.40) | 1.01 (1.00, 1.01) |
| Use | 1 | 5.76 (1.67, 36.4) | 7.12 (2.04, 45.1) | 5.99 (1.36, 41.9) | 1.01 (1.00, 1.03) |
| Glucose-lowering medications | 1 | 1.95 (0.93, 4.61) | 2.12 (1.02, 5.01) | 0.75 (0.25, 2.21) | 1.01 (1.00, 1.02) |
| GLP-1RA | 1 | 3.40 (0.99, 21.4) | 4.33 (1.28, 27.0) | 1.41 (0.29, 10.2) | 1.02 (1.00, 1.03) |
| DPP4i | 1 | 3.90 (0.72, 72.8) | 5.69 (1.05, 106) | 1.25 (0.05, 33.9) | 1.02 (1.00, 1.05) |
| SGLT2i | 1 | 2.33 (0.24, 53.1) | 2.57 (0.25, 60.0) | - | 1.01 (0.96, 1.05) |
| TZD | 1 | 1.27 (0.15, 27.1) | 1.40 (0.17, 29.9) | - | 1.02 (0.99, 1.07) |
| Metformin | 1 | 3.17 (1.06, 13.6) | 4.40 (1.48, 18.9) | 0.42 (0.02, 3.52) | 1.01 (0.99, 1.02) |

Model was adjusted for sex, BMI, blood pressure, LDL-cholesterol, triglyceride levels, eGFR, and the use of antihypertensive, lipid-lowering, glucose-lowering medications, and duration of DM other than the variables for stratification. All *P* for interaction > 0.05.

Supplementary Table 2. Multiplicative interaction between HOMA-IR and HOMA-β in relation to diabetic retinopathy

| **Variable** | **OR (95% CI)** | **P value** | **VIF** |
| --- | --- | --- | --- |
| HOMA-IR (<5 vs ≥5) | 1.02 (1.01,1.03) | 0.005 | 4.50 |
| HOMA-β (≥66 vs <66) | 1.00 (1.00,1.00) | <0.001 | 2.13 |
| HOMA-IR × HOMA-β | - | 0.003 | - |

Model was adjusted for sex, BMI, blood pressure, LDL-cholesterol, triglyceride levels, eGFR, and the use of antihypertensive, lipid-lowering, and glucose-lowering medications.

VIF = variance inflation factor.


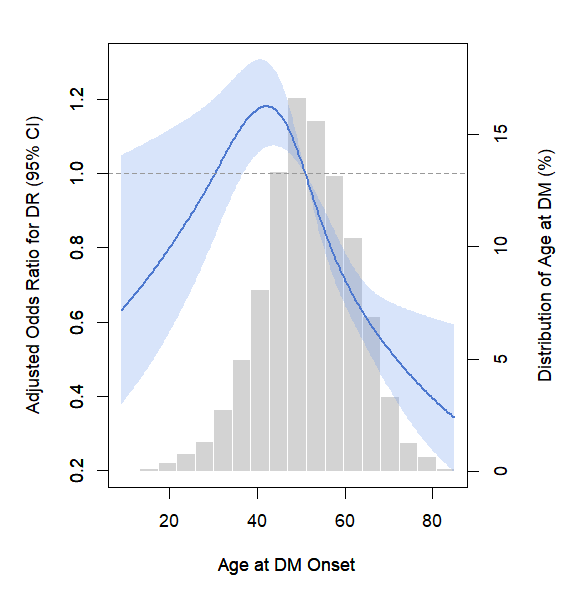


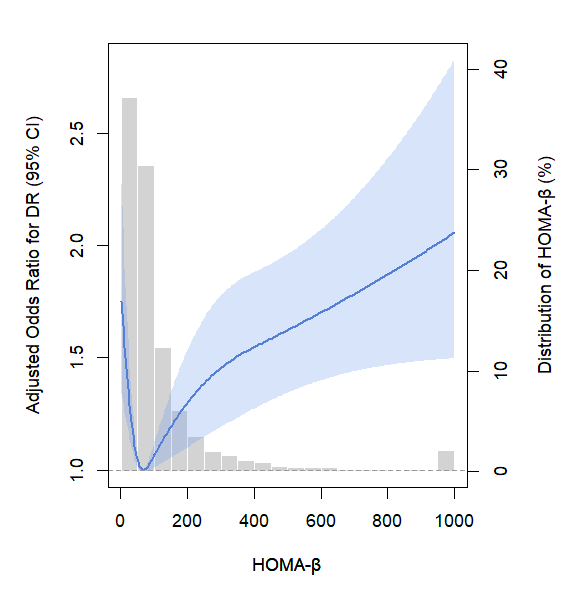


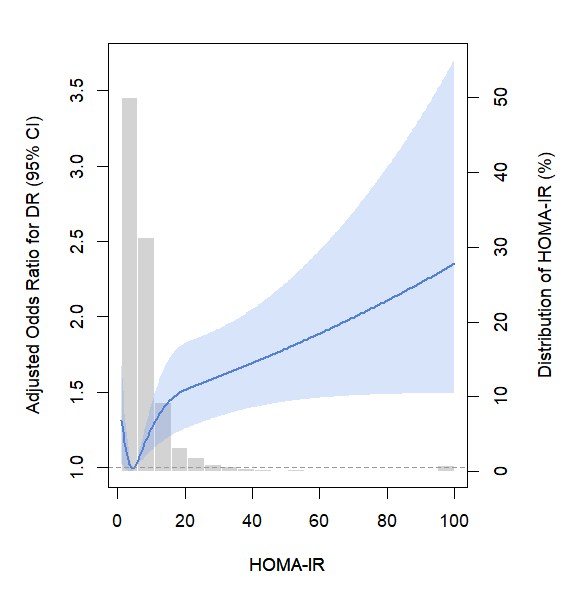

Supplement: Supplementary Table 1 — Subgroup analysis on interactions of odds of diabetic retinopathy among participants with different baseline characteristics of ages at DM onset and HOMA-IR. [file DataSheet1.docx]
